# Genome wide analysis reveals single nucleotide polymorphisms associated with fatness and putative novel copy number variants in three pig breeds

**DOI:** 10.1186/1471-2164-14-784

**Published:** 2013-11-13

**Authors:** Katie E Fowler, Ricardo Pong-Wong, Julien Bauer, Emily J Clemente, Christopher P Reitter, Nabeel A Affara, Stephen Waite, Grant A Walling, Darren K Griffin

**Affiliations:** 1 School of Biosciences, University of Kent, Canterbury, Kent CT2 7NH, UK; 2 Roslin Institute, The University of Edinburgh, Roslin Biocentre, Midlothian, Scotland EH25 9PS, UK; 3 Department of Pathology, University of Cambridge, Tennis Court Road, Cambridge CB2 1QP, UK; 4 JSR Genetics, Southburn, Driffield, East Yorkshirea YO25 9ED, UK

**Keywords:** SNP, CNV, Pig, QuantiSNP, cnvPartition, Fatness, Obesity, Genotyping, GWAS

## Abstract

**Background:**

Obesity, excess fat tissue in the body, can underlie a variety of medical complaints including heart disease, stroke and cancer. The pig is an excellent model organism for the study of various human disorders, including obesity, as well as being the foremost agricultural species. In order to identify genetic variants associated with fatness, we used a selective genomic approach sampling DNA from animals at the extreme ends of the fat and lean spectrum using estimated breeding values derived from a total population size of over 70,000 animals. DNA from 3 breeds (Sire Line Large White, Duroc and a white Pietrain composite line (Titan)) was used to interrogate the Illumina Porcine SNP60 Genotyping Beadchip in order to identify significant associations in terms of single nucleotide polymorphisms (SNPs) and copy number variants (CNVs).

**Results:**

By sampling animals at each end of the fat/lean EBV (estimate breeding value) spectrum the whole population could be assessed using less than 300 animals, without losing statistical power. Indeed, several significant SNPs (at the 5% genome wide significance level) were discovered, 4 of these linked to genes with ontologies that had previously been correlated with fatness (NTS, FABP6, SST and NR3C2). Quantitative analysis of the data identified putative CNV regions containing genes whose ontology suggested fatness related functions (MCHR1, PPARα, SLC5A1 and SLC5A4).

**Conclusions:**

Selective genotyping of EBVs at either end of the phenotypic spectrum proved to be a cost effective means of identifying SNPs and CNVs associated with fatness and with estimated major effects in a large population of animals.

## Background

The study of the process through which pigs convert food comparatively into fat and lean tissue (i.e. the control of fatness) has many potential applications and implications. From an agricultural perspective, pork is the primary source of meat protein worldwide (43%) and global consumption has doubled over the last ten years (World Health Organisation, 2012). The increasing global population and the constant increase in meat consumption in developing countries, especially East and Southeast Asia, bring an unprecedented challenge to the pig breeding industry who are charged, in part, with feeding this growing number of people [1]. Furthermore, market forces demand the tailoring of meats to specific populations and cultures. Pig breeding companies constantly focus their efforts on either producing lean or fat animals or specifically targeted traits e.g. intramuscular fat in their products [2]. While fatness is inexorably linked to diet, genetic factors undoubtedly have an influence and marker assisted selection regimes, aimed at increasing or decreasing growth of fat or lean tissues selectively, require further sophistication. Pork producers aim to improve genetically advanced breeding stock by improving both the food conversion ratio (FCR) and the residual feed intake of each slaughter pig, as well as producing higher yielding carcasses with significant improvement in lean meat percentage [3]. Such sophistication could be advanced further through a deeper understanding of the genetic control of fatness.

The pig is generally considered an excellent model organism for the study of many aspects of human physiology and disease states including cancer, diabetes, [4] maternal aggression [5] and obesity [6-8]. Obesity, excess fat accumulating in the tissues [9], can lead to a variety of illnesses including coronary heart disease, stroke and cancer [10]. Study of the role of genetic variation in the fatness of pigs therefore can have biomedical implications for the understanding and control of one of the biggest killers in the Western world, through the identification of orthologous genes and their variants.

It addition to chromosomal level genomic changes, normal genetic variation at the sequence level includes insertions and deletions (indels) [11], single nucleotide polymorphisms (SNPs) and copy number variants (CNVs). SNPs are the most frequent genetic variation between humans [12]. SNPs are usually biallellic and the least abundant allele (or minor allele frequency (MAF)) usually occurs at in at least 1% of the population [13]. SNPs are commonly found in non-coding regions, however generally do not reside within genes [14]. SNPs act as markers linked to phenotypically relevant loci and they therefore function as powerful tools in non-hypothesis driven research [15]. Certain SNPs have been causally linked with monogenic traits (1 gene/1 trait). Examples of such traits include achondroplasia [16] and sickle cell anaemia [17] as well as variants located near the MC4R gene, which are known to influence fat mass, weight and risk of obesity, with mutations of this gene being a cause of monogenic severe childhood onset diabetes). It is the association of SNPs with polygenic and multifactorial traits however, that has received the most attention in recent years. That is, post-genomic technologies such as SNP microarrays have permitted genome wide association studies (GWAS) for thousands of traits in humans. Such studies include research into complex diseases in humans such as breast cancer [18], type 2 diabetes (T2D) [19,20], Crohn’s disease [21,22], Parkinson’s disease [23], coeliac disease [24] and obesity. Some examples of obesity GWAS in humans include research into early onset extreme obesity. One particular study provided proof of principle for the concept that GWAS are useful in detecting genes relevant to complex phenotypes. In this case a human SNP array comprising of 440,794 SNPs from 487 extremely obese young German samples and 442 healthy lean German controls. Fifteen significant SNPs were determined, 6 of which were associated with the FTO gene, suggesting that it strongly contributes to early onset obesity [25]. To date however, the studies of SNPs associated with fatness in pigs have been limited in comparison to the number of human studies [26-28]. Indeed, there are few GWAS in the pig in relation to phenotypic traits. Popularly studied traits in the pig, apart from fatness include boar taint [29-31], body composition and structural soundness [32,33], however such studies are also still in their infancy; this perhaps reflects the fact that a SNP microarray (chip) for the pig has only been developed within the last 4 years. Although these studies have provided much insight into the molecular biology of many traits, more are necessary in order to fill gaps in our knowledge of fatness, as it is a multifactorial, complex trait. The agricultural sector stands to benefit financially from such research and there are potentially biomedical applications if orthologous human genes can be associated with obesity, especially if GWAS can be performed in a low-cost manner by means such as use of selective genotyping. Selective genotyping, i.e. use of individuals at the extreme ends of a phenotypic spectrum provides an effective means of performing GWAS on a large population by sampling small numbers of animals and has its theoretical basis in the early 1990s [34]. Most recently applied to identify SNPs associated with back fat thickness in pigs used for Italian dried ham [35].

CNV regions (CNVRs) are segments of DNA (ranging from 1 kilobase (kb) to several megabases (Mb) in size [36]) that vary in copy-number by comparison with reference genomes [36]. It is thought that 12% of the human genome contains CNVRs [37] that are heritable in normal individuals. Around 0.4% of genome content in people who are unrelated is thought to vary in copy number [38]. Specific algorithms for CNV detection from SNP chip raw data include ‘PennCNV’, ‘GADA’ [39], ‘CONAN’ [40], ‘cnvPartition’ and ‘QuantiSNP’, the last 2 of which have been used in this study. Comparative analyses of the above have suggested a preference for QuantiSNP [41]. The study of CNVs has been pioneered in humans and catalogues of human CNVs are now available [42-44]. Significant associations of CNVs with human disease are numerous as CNVs are thought to be able to influence gene expression and may also play a role in affecting metabolic traits. These include susceptibility to HIV1 [45], a role in auto immune disease and lupus glomerulonephritis [46]. CNVs are strongly correlated with gene, repeat and segmental duplication content [47] and play a significant role in the development of complex traits. A recent study showed that 19 CNVs are significantly associated with the mechanisms for the control of a number of metabolic traits in mice. Indeed mouse chromosomes 1, 4 and 17 all have CNVRs in regulatory regions influencing body weight [48]. Wang et al. [49] used PennCNV on SNP chip data in humans to study obese individuals with ‘never overweight’ controls. The authors determined that in a study of over 800 individuals, large CNVs and rare deletions were associated with the risk of moderate obesity [49]. Moreover, CNVs contained candidate obesity genes including a 3.3 Mb deletion disrupting NAP1L5 (nucleosome assembly protein 1-like 5) as well as a 2.1 Mb deletion disrupting UCP1 (uncoupling protein 1) and IL15 (interleukin 15). Such studies for the determination of CNVRs in pigs however are, to date, limited to only 6. The first focussed on chromosomes 4, 7, 14 and 17, in which 37 CNVRs were identified [50], with the second identifying 49 CNVRs genome wide [51]. The third, published in 2012, used a sample size of 474 pigs, across 3 pure-bred lines, Yorkshire, Landrace and Songliao Black as well as a cross-bred line, Duroc-Erualian. PennCNV was the chosen platform, with 382 CNVs identified, genome wide, from information from the Illumina SNP60 platform [52]. Chen and colleagues also used PennCNV to analyse porcine CNVs; they found 565 CNVRs containing a total of 1315 CNVs, from 18 populations. Hotspots for these CNVs included areas on chromosomes 6, 11, 13, 14 and 17, with the Duroc breed having the most CNVs found per individual [53]. A study in 2012 using array comparative genomic hybridisation (CGH) on 12 pigs from several different breeds (including Large White and Duroc) highlighted 259 CNVRs [54], whilst Wang and colleagues used PennCNV to glean CNV information from data derived from the Illumina Porcine SNP60 Beadchip, finding 382 CNVs in a total number of 474 pigs [52].

Studies in the pig have not before however, studied the effect of CNVs on specific traits, with few using QuantiSNP or cnvPartition.

Given the above, it is clear that, despite the need to understand the genetic control of fatness in pigs, both from an agricultural and biomedical standpoint, very few genes have hitherto been associated with fatness and/or leanness in this species. The availability of the porcine 60 K SNP chip (Illumina) allows us to ask whether there are SNPs and/or CNVRs significantly associated with defined fatness phenotypes in different pig breeds. To date, we are aware of only one GWAS and no CNV searches that have addressed this question. Fontanesi and colleagues [35] looking at a single breed and finding gene ontology terms associated with nervous system development and regulation. With this in mind, in this study, we performed a GWAS using selective genotyping to sample a population of over 70,000 animals from 3 breeds identifying both significant SNPs and extracting quantitative information from the raw data to identify also CNVs.

## Results

### SNP analysis

**Figure 1:**
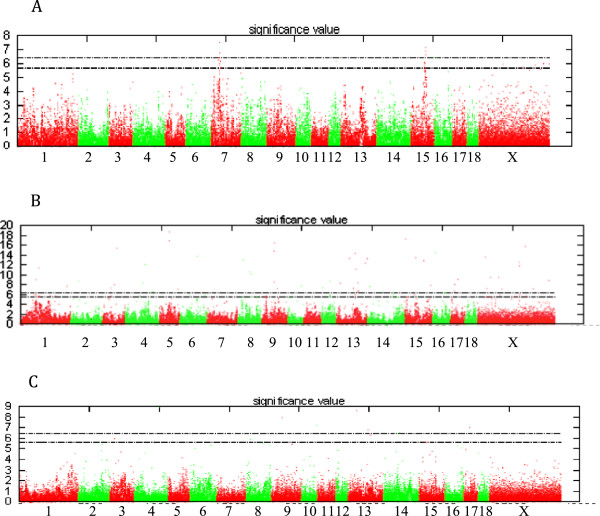
**Graphical interpretation of SLLW (A), Duroc (B) and Titan (C) SNP results for Rib Fat for all chromosomes.** Legend: Graphical interpretation of SNP results for Rib Fat for all chromosomes. Red dots represent SNPs on odd numbered chromosomes while the green dots are from even numbered chromosomes. The dotted lines represent the 5% (bottom line) and 1% (top line) confidence thresholds.

**Figure 2:**
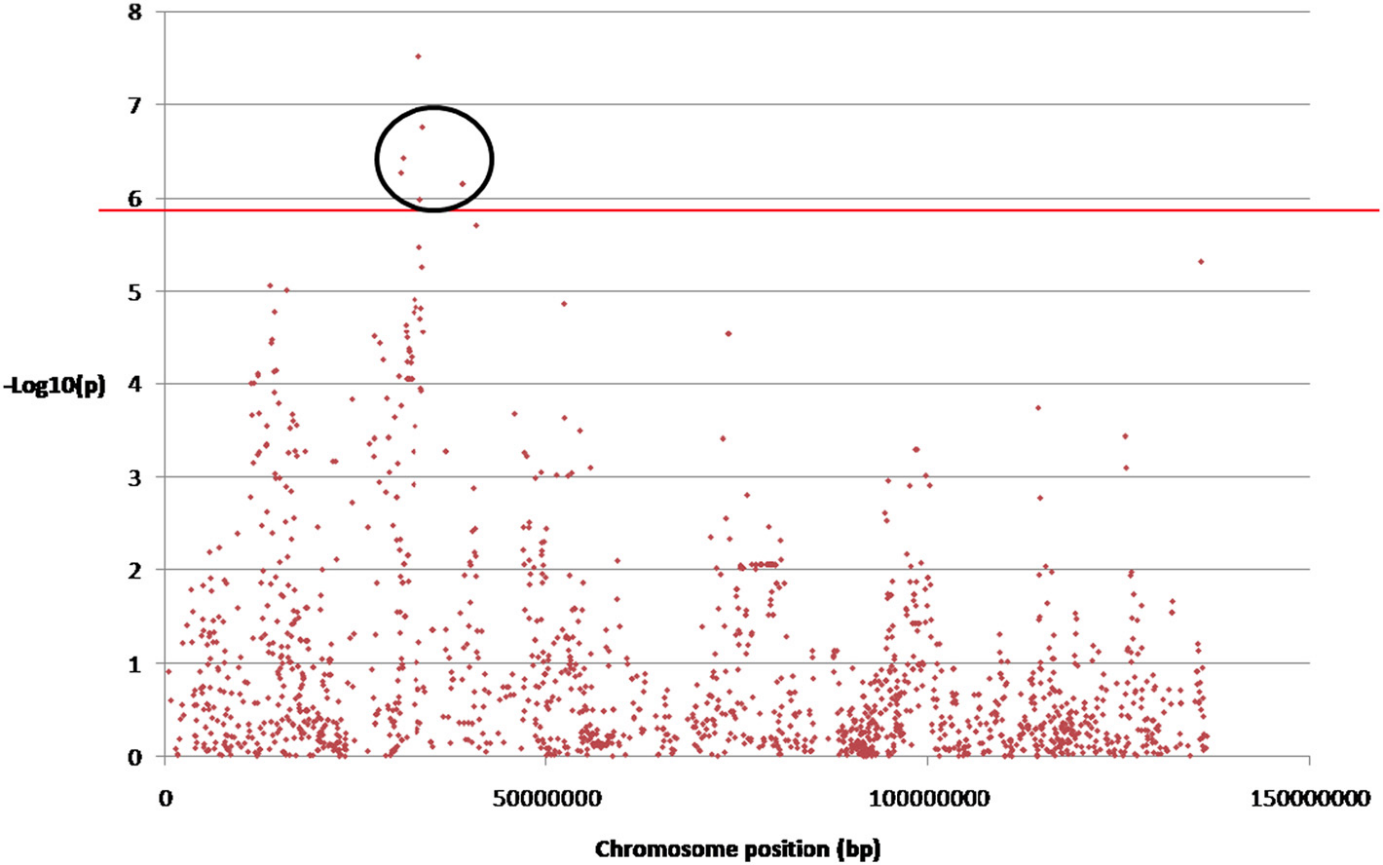
**Graphical microarray outputs from chromosome 7 in the SLLW breed.** Legend: Graphical microarray outputs from chromosome 7 in the SLLW breed. Each of the dots represents a SNP plotted against its chromosome position (bp) and significance value (-log10(p)). The circled SNPs represent SNPs discovered within the following genes: TINAG, DST and GLO1.

Selective genotyping permitted the generation of statistically significant results using Estimated Breeding Values (EBVs) from animals in the upper and lower 5th percentiles. After SNP chip interrogation, a total of 33,080 SNPs were eliminated from the analysis from the original 64,232 due to either the Minor Allele Frequency (MAF) being <0.05, SNPs being located on the sex chromosomes or those that deviated from Hardy Weinberg Equilibrium (HWE). 31,152 SNPs were taken further for analysis. The results shown in Figure 1 (A, SLLW, B Duroc and C Titan) are the Manhattan plots obtained for the 3 breeds. This figure displays the results from an additive model with significant SNPs at the 5% genome wide level; (p value greater than 0.000019952 (i.e. –log10(p) equal to 5.7)) (see Methods). An example of a chromosome that had significant clustered SNPs present in chromosome 7 (SLLW breed) is shown in Figure 2. Clustering of other SNPs near a SNP of interest reinforces its significance. For the 3 breeds, Duroc, Titan and SLLW, a total of 50, 10 and 12 SNPs (respectively) were considered to be significant, all of these being breed specific. The most significant SNPs were located on chromosomes 7 and 15 (SLLW), 5 and 15 (Duroc) and 9 and 13 (Titan). Following gene ontology, genes that either had an association with fatness (4 genes) or those that resided within a gene (17 genes) are shown in Table 1. Table 2 shows SNPs that were deemed as significant in this study, but without genes upstream or downstream that were associated with fatness. Significant SNPs that were located either up or downstream from genes and implicated in the fatness phenotype in the Duroc breed, were MARC00776967 downstream from NTS on chromosome 5 (neurotensin, involved in maintenance of gut structure and function, and in the regulation of fat metabolism), ASGA0073794 upstream from FABP6 on chromosome 16 (FABPs roles include fatty acid uptake, transport, and metabolism), INRA0040972 upstream from SST on chromosome 13 (somatostatin, an important regulator of the endocrine system through its interactions with pituitary growth hormone, thyroid stimulating hormone, and most hormones of the gastrointestinal tract) and ASGA0102890 upstream from NR3C2 located on chromosome 8 (which encodes the mineralocorticoid receptor, involved in metabolism). 12 other significant SNPs identified in the Duroc breed, resided in the genes TECTA, EPAS1, TMPRSS4, ADAM32, MX2, HSF5, MPZL3, CAMK1D, DOCK5, CCDC39, DENND2D and TEX14. Only 1 significant SNP (ALGA0027483) was found to be located within an intronic region of a gene for the Titan breed. This gene was SPAG17, known to be involved in maintenance of the structural integrity of the central apparatus of the sperm axoneme. There were no genes implicated with fatness found in the Titan breed. Significant SNPs in the SLLW breed were identified in 3 chromosomes, 7, 15 and 16. Whilst there were no genes identified either up or downstream from significant SNPs, that could have been directly implicated with a fatness phenotype, other significant SNPs that resided within genes included ALGA0039880 in the gene TINAG (tubulointerstitial nephritis antigen), INRA0024695 and ALGA0040094 both in the gene DST (dystonin) and DIAS0000554 in the gene GLO1 (glyoxalase I). The only gene that had a SNP located within a coding region in any of the 3 breeds was GLO1. After analysis using the Ensembl variant effect predictor, the amino acid at position 41 displayed a synonymous substitution, with the residue leucine not being modified after the SNP change. All significance levels for all SNPs in all breeds are depicted in Figure 1. It was interesting to note that the Duroc breed had many more SNPs associated with a fatness phenotype than any of the other breeds analysed with these SNPs having a higher level of significance also.

**Table 1:** Significant SNPs and genes implicated in this study

**Chromosome**	**Position**	**Gene**	**SNP position**	**Locus**	**SNP code**	**Breed**	**Fat/Lean**
5	91141085	NTS	Downstream	MARC0076967	A/C	Duroc	Rib fat
16	61040142	FABP6	Upstream	ASGA0073794	A/G	Duroc	Rib fat
13	94991811	SST	Upstream	INRA0040972	A/C	Duroc	Rib fat
8	69607171	NR3C2	Upstream	ASGA0102890	A/G	Duroc	Rib fat
9	46808866	TECTA	In	ASGA0086155	A/G	Duroc	Rib fat
3	87581931	EPAS1	In	DRGA0004073	A/C	Duroc	Rib fat
9	44294242	TMPRSS4	In	ASGA0096348	A/C	Duroc	Rib fat
15	44784671	ADAM32	In	ALGA0085262	A/C	Duroc	Rib fat
13	144382945	MX2	In	ALGA0074022	A/G	Duroc	Rib fat
12	32729466	HSF5	In	ASGA0054362	A/G	Duroc	Rib fat
9	44430920	MPZL3	In	CASI0012011	A/G	Duroc	Rib fat
10	56352838	CAMK1D	In	M1GA0014251	A/C	Duroc	Rib fat
14	9438426	DOCK5	In	ALGA0075006	A/G	Duroc	Rib fat
13	90285880	CCDC39	In	ALGA0071824	A/C	Duroc	Rib fat
4	113559620	DENND2D	In	M1GA0006409	A/G	Duroc	Rib fat
12	32843547	TEX14	In	ALGA0066216	A/G	Duroc	Rib fat
4	106837043	SPAG17	In	ALGA0027483	A/C	Titan	Rib fat
7	30950588	TINAG	In	ALGA0039880	A/G	Sire line	Rib fat
7	33345033	DST	In	INRA0024695	A/G	Sire line	Rib fat
7	33530081	DST	In	ALGA0040094	A/G	Sire line	Rib fat
7	39122530	GLO1	In	DIAS0000554	A/C	Sire line	Rib fat

Table shows the position of significant SNPs either associated with genes implicated in a fatness phenotype (grey) and SNPs within genes, discovered through the interrogation of the Illumina SNP60 BeadChip. The chromosomal position, position of the SNP in relation to the gene (located either within the gene (denoted as ‘in’), downstream or upstream), SNP code and the breed in which the gene was identified are also shown.

**Table 2:** Table shows SNPs identified as being significant in this study and genes (not associated with fatness) located 0.5 Mb upstream or downstream from each SNP, discovered through the interrogation of the Illumina SNP60 BeadChip

**Duroc**		
**SNP ID**	**Genes upstream**	**Genes downstream**
INRA0003205	SH3BGRL2, ELOVL4, TTK	LCA5, HMGN3
INRA0002667	-	MANEA
ALGA0010375	STRBP, GPR21, ZBTB6, RC3H2	CRB2, DENNDIA
MARC0026060	-	-
MARC0051272	-	-
INRA0005757	IFNB1, PTPLAD2, KIAA1797	MLLT3
ALGA0006852	NOX5	GLCE, KIF23, PAQR5
ASGA0003731	TTK, ELOVL4	SH3BGRL2
DRGA0002899	BBOX1, CCDC34, LIN7C, LGR4	SLC5A12, ANO3, MUC15
ALGA0019845	SLC1A4, RAB1A, CEP68	SERTAd2, AFTPH
ASGA0014296	PHF2, FAM120A	BARX1, PTPDC1
MARC0074041	UXS1, NCK2	FHL2
MARC0068661	FAM78B	UCK2
INRA0014911	TOX	-
MARC0005831	-	PEX2
ALGA0031998	-	-
ALGA0031986	-	PLEKHA5
DRGA0006714	DSC1	RNF125
ASGA0095121	GLTPD1, TTLL10	MIB2, CDK11A
ALGA0117693	EFS15, RNF11, TTC39A, CDKN2C	-
H3GA0024542	-	-
MARC0045989	CNOT6L, MRPL1	FRAS1, NPY2R
DIAS0001163	-	-
ALGA0115519	-	PHF14
MARC0022409	PAK1	INTS4, KCTD14, ALG8
MARC0001591	CD300LB, RAB37, NAT9, GRIN2C, FADS6	-
MARC0016326	RNF43, BZRAP1	SEPT4, TEX14
ALGA0066217	RAD51C	TRIM37
ALGA0066214	SUPT4H1, RNF43, BZRAP1	MTMR4, SEPT4, TEX14
ASGA0058594	RAP2B	P2RY1
H3GA0036520	RYBP	EIF4E3
ALGA0073528	-	C21orf7, BACH1
MARC0032428	GOLIM4	MECOM
ASGA0102446	PTX3, VEPH1	SHOX2, RSRC1
ALGA0122006	CD47, IFT57, HHLA2, MYH15	KIAA1524, DZIP3, RETNLB
DRGA0012091	-	RBMS3
ALGA0070937	LRRTM4	-
INRA0040988	OSTN, UTS2D, CCDC50	-
DRGA0012752	MBNL1	P2RY1
ALGA0109563	-	-
H3GA0040313	NID1, GPR137B, ERO1LB	GNG4, TBCE, B3GALNT2, GGPS1
ALGA0080178	MBL2	DKK1, PRKG1
ALGA0080086	-	-
ALGA0083515	-	-
ASGA0070192	MYO1B	OBFC2A, SDPR
MARC0015113	COPS7B, PDE6D, NMUR1, NCL	D1S3L2, ECEL1, CHRND, CHRHG
ALGA0085117	-	-
ASGA0095282	-	-
ALGA0094547	IDH3B, TMC2	STK35, PDYN
ASGA0075751	-	SPTLC3, TASP1
ASGA0075610	PLCB1, PLCB4	-
**SLLW**		
SNP ID	Genes upstream	Genes downstream
ALGA0039930	LRRC1	C6orf142
ALGA0040140	COL21A1	-
ALGA0040529	GLO1	DNAH8
ASGA0032973	-	-
DRGA0015258	ATP5G3	-
ASGA0070042	ATP53G	-
ALGA0086180	-	HOXD11
MARC0049797	CCDC141	SESTD1
ASGA0070135	SESTD1	-
INRA0049850	SSFA2	PPP1R1C
ASGA0070147	DNAJC10	-
ALGA0115880	-	-
MARC0085425	-	AGPS
**Titan**		
SNP ID	Genes upstream	Genes downstream
ASGA0014024	-	ABCC1
MARC0044718	-	-
ASGA0042741	FAM55D	-
ASGA0048928	PRKCQ	SFMBT2
MARC0069967	LARS2	-
DRGA0012769	MME	GMPS
ASGA0058709	-	TBL1XR1
DRGA0013970	RHOBTB1	-
ALGA0109681	GYPC	-
MARC0078259	-	DTD1

### CNV Analysis

**Figure 3:**
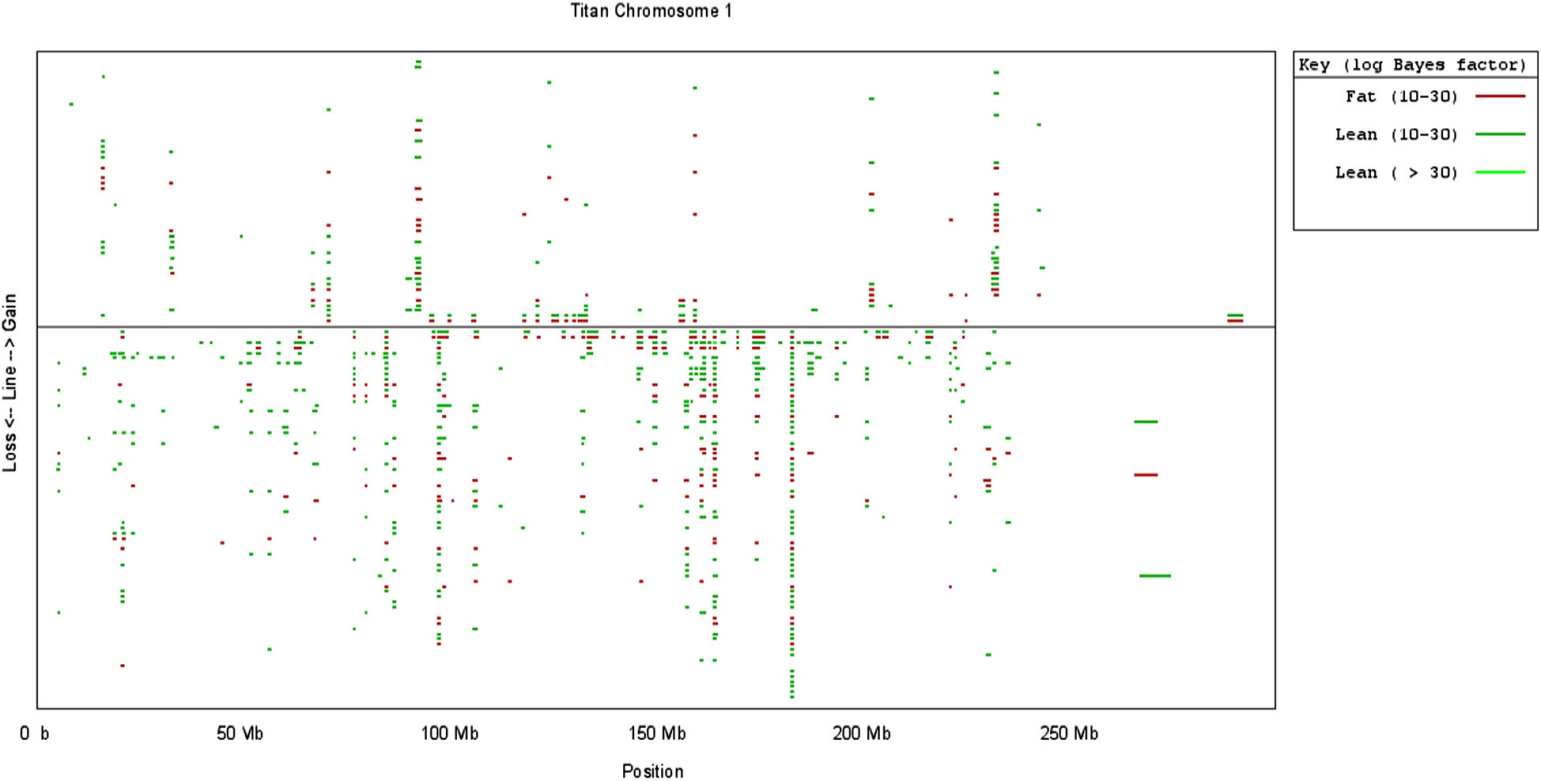
**QuantiSNP output for Titan chromosome 1.** Legend: QuantiSNP output for Titan chromosome 1. Red lines depict CNVs discovered in fat pigs and green lines depict those found in lean pigs. To distinguish between levels of significance (determined by Log Bayes Factors), a darker line (either red or green, as shown in the key) indicates a high Log Bayes Factor (>30), and therefore a higher level of significance, while a paler line indicates samples with a Log Bayes Factor of between 10 and 30 (therefore with a lower significance value). Lines above the x-axis in the middle of the figure, indicate gains in copy number whilst lines below are losses. The chromosome position in Mb is shown below the graph and the y axis is scaled by copy number count.

In terms of CNV Analysis, 1 example of the output from QuantiSNP is shown in Figure 3 for Titan chromosome 1. All red lines on this figure depict CNVs discovered in ‘fat’ (upper 5th percentile) pigs and green lines depict those found in ‘lean’ (lower 5th percentile) pigs. In order to distinguish between levels of significance (determined by Log Bayes Factors), a darker line (either red or green, as shown in the key) indicates a high Log Bayes Factor (>30), and therefore a higher level of significance, while a paler line indicates samples with a Log Bayes Factor of between 10 and 30 (therefore with a lower significance value). Lines above the x-axis in the middle of the figure, indicate gains in copy number whilst lines below are losses. The chromosome position in Mb is shown below the graph. The start and ends of the putative CNVs are defined on the chip and through reference to the published porcine genome sequence. The total number of CNVs detected was higher from QuantiSNP (216) than from cnvPartition (27). Of the total 243 CNVs detected in 5 or more animals (from either CNV calling approach), 202 were unique to QuantiSNP (83%), 14 were unique to cnvPartition and 27 were detected by both (11%). Generally, there were 3 times more losses found by both programs than gains. In order to determine whether regions of the genome differ in DNA copy number in statistically different numbers of ‘fat’ and ‘lean’ animals, Mann Whitney U tests (2 tailed) were performed; samples with a p value of ≤0.05 were considered significant. The statistical results for significant CNVRs can be found in Table 3, that shows CNVs present in more than 5 animals, for both CNV detecting algorithms. The estimated copy number and the number of animals in which the CNVs were found is shown. Each CNV was assigned a CNV ID; 1.S2 represents a CNV on chromosome 1, from the SLLW breed, assigned the number 2. 2 CNVRs contained genes that their ontology suggested could play a role in fatness, shown in Table 4. These CNVs were 5.D2 (chromosome 5, Duroc) and 14.D1 (chromosome 14, Duroc). 98% of the CNVs overlapped coding sequence. In the SLLW breed, cnvPartition results covered 6.11% of the genome, while QuantiSNP determined a 11.26% coverage. cnvPartition and QuantiSNP results for the Duroc breed indicated genome coverage of 5.03% and 10.93% respectively, with the percentage coverage in the Titan breed being 4.22% (cnvPartition) and 0.62% (QuantiSNP). The distribution of the CNVs can be found in the Additional file 1: Figure S1. Here, the positions of the CNVs are noted with respect to a standard porcine ideogram (ignoring whether these animals are fat or lean). It is depicted whether the CNVs (found in 5 or more animals) are losses or gains by the position, left or right of the chromosome respectively, in which breed they were observed (colour code) and in how many animals each CNV was observed (number inside the coloured ellipse). Results from both QuantiSNP and cnvPartition are given side by side.

**Table 3:** Significant CNVR results comparing fat and lean samples using Mann Whitney (two tailed) testing

**Program**	**CNV ID**	**Chromosome**	**Start position**	**End position**	**Copy number**	**Fat**	**Lean**	**p(2)**	**U**
cnvPartition	14.S1	14	49996213	53700421	0	29	18	0.05	1416
					1	0	0		
					2	19	30		
					3	0	0		
					4+	0	0		
cnvPartition	6.D1	6	68258924	78889296	0	27	16	0.05	1416
					1	0	0		
					2	21	32		
					3	0	0		
					4+	0	0		
cnvPartition	14.D1	14	50332490	53700421	0	29	40	0.05	888
					1	0	0		
					2	19	8		
					3	0	0		
					4+	0	0		
QuantiSNP	4.D3	4	79881	2580313	0	0	0	0.01	768
					1	28	44		
					2	20	4		
					3	0	0		
					4+	0	0		
QuantiSNP	4.D5	4	74926691	77748130	0	0	0	0.05	888
					1	28	39		
					2	20	9		
					3	0	0		
					4+	0	0		
QuantiSNP	5.D2	5	33971	9428425	0	0	0	0.003	744
					1	23	40		
					2	25	8		
					3	0	0		
					4+	0	0		
QuantiSNP	14.D1	14	47658364	58439670	0	0	0	0.01	768
					1	12	28		
					2	36	20		
					3	0	0		
					4+	0	0		
QuantiSNP	15.D2	15	90602719	95659643	0	0	0	0.05	888
					1	29	40		
					2	19	8		
					3	0	0		
					4+	0	0		
cnvPartition	14.T2	14	74855162	77603365	0	19	36	0.003	744
					1	0	0		
					2	29	12		
					3	0	0		
					4+	0	0		
cnvPartition	15.T1	15	19895180	21543806	0	4	18	0.01	816
					1	0	0		
					2	44	30		
					3	0	0		
					4+	0	0		
QuantiSNP	1.T2	1	18418830	18641391	0	0	0	<0.0001	456
					1	17	46		
					2	31	2		
					3	0	0		
					4+	0	0		
QuantiSNP	4.T5	4	43206823	45758968	0	0	0	0.05	1416
					1	0	0		
					2	43	32		
					3	5	16		
					4+	0	0		

Table shows significant CNVs (determined by Mann Whitney U (two tailed) tests, present in more than 5 animals for both QuantiSNP and cnvPartition. The chromosome and start and end of each of the CNVRs, is given. The estimated copy number of the sequence and the number of animals in which each was observed is also provided. Each CNV was assigned a ‘CNV ID.’ For example ‘1.S2’ denotes that the CNV is on chromosome 1, it is from SLLW breed and it has been assigned the number ‘2’. Duroc and Titan CNVs were assigned the letter D and T respectively.

**Table 4:** All genes significantly associated with fatness that are contained between the estimated start and end of the CNVRs identified in this study

**CNV ID**	**Breed**	**Chromosome**	**Gene CODE**	**Gene name**	**Function**
5.D2	Duroc	5	MCHRI_PIG	Melanin concentrating hormone receptor 1	Regulates functions in the mammalian brain, particularly feeding behaviour; mice lacking MCH eat less and are therefore lean
5.D2	Duroc	5	F1SM76_PIG	Peroxisome proliferator activated receptor alpha	Involved in metabolic control of the expression of genes encoding fatty acid oxidation
14.D1	Duroc	14	F1RLV1_PIG	Sodium/glucose transporter	Mediates transport across cellular membranes responsible for active glucose absorption across brush border of cells that lie in the gastrointestinal tract
14.D1	Duroc	14	F1RLU7_PIG	Sodium/glucose co-transporter	Low affinity sodium/glucose transporter

## Discussion

The results presented demonstrate the applicability of selective genotyping when using a GWAS approach. Unlike the most recent similar study [35] the breeds under investigation are ones marketed extensively worldwide and clearly display breed specific differences. For GWAS data to be used in practical applications, it is essential that the SNPs discovered as associated with fatness and leanness (or indeed any other commercially relevant trait) are segregating in the population in question. To this end, in any association study, the benefits must outweigh the costs; here we have provided evidence of a low-cost approach to GWAS by using EBV data for animals in the upper and lower 5th percentiles. It is also essential that the relevant samples are archived and readily accessed. Storage of large numbers of blood or DNA samples in freezers is expensive and space consuming. Here we demonstrate that amplifying DNA from archived blood spots can overcome this problem. Finally, we demonstrate that CNV information can be derived from the raw SNP chip data, providing the opportunity for studies of DNA copy number and its association with commercially and biologically relevant traits.

### SNP discovery and gene ontology

In the current study, we identified a total of 12 SNPs in the SLLW breed, 50 in Duroc breed and 12 in Titan breed significantly associated with fatness or leanness as defined by EBVs for back fat. The reasons why Duroc had so many more than the other two (indeed over twice as many as the other two put together) is not clear. We are not aware that Duroc is any more genetically diverse – one possible explanation is a technical one in that the chip itself was made from a Duroc pig and the results may reflect ascertainment bias. SNPs were identified that were either contained within, or 0.5 Mb up or downstream of genes whose ontology terms strongly implicated them in a fatness phenotype as follows: NTS (neurotensin), is involved in energy homeostasis, a complex physiological process most probably related to fatness [55]. Genes such as NTS have been shown to be involved in both central and peripheral signals affecting feed intake [56,57]. SST (somatostatin) was first isolated from the hypothalamus of sheep as a 14 amino acid peptide in 1973 [58]; SST plays a vital role in the regulation of growth and development in vertebrates, particularly muscle growth. It is known to be one of the most important genes involved in both the regulation of animal growth, metabolism and development through its negative role on growth, as it acts as an inhibitor of growth hormone release [59], inhibition of cell proliferation as well as affecting nutrient absorption in the alimentary canal [60,61]. A study focussing on a polymorphism in SST and its association with growth traits in Chinese cattle was published earlier this year that has been correlated with improving the establishment of meat production performance by breeding of new beef cattle [58]. Fatty acid binding protein 6 (FABP6), which was mapped to chromosome 16 in 2007 [62], was also found to be significant in the Duroc population in this study. It has been shown that FABP6 is associated with type II diabetes therefore suggesting an association of variants between fatness and type II diabetes susceptibility [63], as well as the role of FABPs in fat absorption and in the development of metabolic syndrome [64]. GLO1 (SSC17) was a gene of particular interest highlighted throughout this study due to the fact that it was the only candidate gene isolated in this GWAS that had a SNP residing within the gene itself. GLO1 is a candidate gene that is thought to be involved with fatness; a study that focussed on a QTL for carbohydrate and total energy intake on chromosome 17 in mice showed that genes (including GLO1) involved in fat metabolism were decreased in carbohydrate preferring mice [65,66]. A GWAS performed in 2009 discovered specific alleles that interestingly were associated with both increased back fat and better leg action [67]. These genes included MTHFR, WNT2, APOE, BMP8, GNRHR and OXTR.

The results presented have also been compared to a recent GWAS performed in a specific sire line large white breed used in dried Italian ham production [35]. The genes which Fontanesi and colleagues found to be associated with significant SNPs by gene ontology, did not overlap with ones found in this study, however interesting insights were made, possibly indicating that neuronal genes may play a role in fat deposition in the pig. This concurs with one of the genes we found to be associated with fatness, neurotensin, widely distributed throughout the central nervous system that may be a neuromodulator or neurotransmitter.

### Technical issues pertaining to GWAS

There are several factors to consider when interpreting GWAS for example, in replication of such studies, the consistency of the results vary [68]. Some genes are reproducibly found in follow-up studies, such as genes related to diabetes including the peroxisome proliferator-activated receptor-γ (PPARG). This was of particular interest as the peroxisome proliferator activated receptor alpha, was found in a CNV which was significant in this study. Replication problems involved in replication of GWAS have been widely reported [69-72] also mentioning that small sample sizes can be problematic. Inconsistent results have been reported in obesity studies [69], which suggests that many results may be population dependent.

One possible criticism of the results presented here is the relatively small sample size used. We would argue that this might have been more of a problem had we considered the data as a binary trait (i.e. either ‘fat’ or ‘lean’). We believe that there is no loss of power (when compared to analysing the entire population of over 70,000 animals, from which the EBVs were derived) by adopting selective genotyping, indeed we suggest that use of this approach (i.e. using EBVs from animals in the tails of the distribution), in fact, retains most of the power of the calculations. If the EBVs are calculated from a larger number of individuals then it removes a source of environmental variation and potentially provides a more accurate estimate of the genetic effect (taking into account information from other animals in the population that were not genotyped). As Darvasi and colleagues showed, selective genotyping is very effective to retain the power of the experiment while reducing the cost of genotyping [34]. There is no evidence that selective genotyping would bias results, thereby increasing the number of false positives, providing that an appropriate significance threshold is calculated to account for multiple testing. A similar approach was recently published in this journal by Fontanesi and colleagues [35] have demonstrated that selective genotyping is very effective to retain the power of the experiment while reducing the cost of genotyping. Given this information, we were able to derive data from a 70,000+ animals, thereby avoiding a high false positive rate. Admittedly we might miss QTL with relatively small effects, however part of the point of the exercise was to discover traits that were most biologically significant and thus commercially relevant.

### CNV analysis

The CNV information presented is, one of few such studies in pigs and has identified 243 candidate CNVs. It is worthy of note however that further studies involving deep sequencing, array CGH and or qPCR would be required to confirm the extent to which the data presented here represent physical changes on DNA copy number. Nonetheless we have demonstrated that use of SNP chips can identify “putative” CNVs that warrant further investigations. Our attempts to confirm some of the results are mentioned below. Only 2 putative CNVs that were discovered contained genes that, as implied by their ontology terms, might be involved in fatness. Both programs identified CNVR14.D1 whereas QuantiSNP only called CNVR5.D2. In CNVR5.D2, a copy number loss (in comparison to the reference genome) was observed in both fat and lean animals, however the number of animals that displayed a loss was significantly greater in lean animals (40) compared to fat animals (23). This is particularly interesting, due to the fact that 1 of the genes located within this putative CNVR was MCHR1, known to regulate feeding behaviour. It has been shown that mice lacking MCHR1 eat less and are therefore leaner [73,74]. PPARα, involved in metabolic control of the expression of genes encoding fatty acid oxidation enzymes [75], is also located in this putative CNVR. CNVR14.D1, again displayed a greater number of samples with a loss in copy number in ‘lean’ compared to ‘fat’ animals (28 vs 12). Genes contained within this CNVR include sodium/glucose transporters and co-transporters, responsible for glucose absorption across the brush border of gastrointestinal tract cells; similar gene families have also been identified in other pig CNVR studies [52,53]. When comparing the data produced from this study to other published work, there were 7 losses identified by QuantiSNP that were also found by Fadista et al. [50]. Seven overlapping CNVRs were also found when comparing our data to Ramayo-Caldas et al. [51], however there were discrepancies in calling whether these were gains or losses.

A total of 83% of the 243 putative CNVs identified were unique to QuantiSNP, 6% were unique to cnvPartition and 11% were detected by both programs. One possible reason for these discrepancies is that each of the programs made use of a different algorithm in order to detect putative CNVs. cnvPartition, the plug-in available for Illumina’s Genome Studio, produces 1 of 14 possible outputs assuming 5 copy number states; a homozygous deletion, heterozygous deletion, dizygous (normal state), trizygous (1 extra copy) and finally tetrazygous (2 more copies than the normal state). This algorithm models log *r* ratios (LRRs) and b allele frequencies (BAFs) as a bivariate Gaussian distribution. In contrast, QuantiSNP is based on a Hidden-Bayes Objective Markov Model (HB-OMM) that considers the number of copies in both the hidden and observed states. QuantiSNP uses a filtering process by which any CNVs called with a Log Bayes Factor of less than ten were removed whereas cnvPartition does not. Taking this into consideration it is surprising that QuantiSNP called more CNV regions than cnvPartition, however this could suggest that the algorithm used by the cnvPartition software is more accurate at calling CNVs. Generally, there were more losses found by both programs than gains. A paper published this year discussed new freely available software called ParseCNV. This is a CNV call association software that uses CNV information to create SNP statistics from information in the PennCNV format [76]; this would be an interesting future study to perform on this data.

qPCR was attempted in order to verify the results produced from both cnvPartition and QuantiSNP, however, after numerous attempts, results did not give adequate consistency in order to calibrate the system and therefore make it possible to verify the putative CNV calls by independent means. As discussed in a study published in 2010, there are several reasons as to why CNVR prediction varies between qPCR analysis and *in silico* analysis of data. The 4 x sequence depth of the *Sus scrofa* genome build 9 and low probe density of the Porcine SNP60 BeadChip makes it difficult to determine the genuine boundaries of CNVRs. This can therefore lead to an over-estimation of the real size of the region. SNPs and indels also have the ability to affect the hybridisation of qPCR primers and true CNVR boundaries may be polymorphic between analysed animals [51]. Array CGH could also be used as an alternative platform to derive CNV information from and compared to data produced in this study. There have been several such studies performed [37,77,78] including one in pigs [51]. Whilst this would be an interesting comparison, it is possible that similar amplification problems might be experienced.

## Conclusions

The combination of a cost effecting selective genotyping-based GWAS, data from 3 widely consumed pig breeds, the derivation of data from archived blood spots and the simultaneous mining of both SNP and CNV data are the unique aspects of this study. The discovery of novel SNPs and CNVs associated with fatness are, potentially of value to the pig breeding industry and shed light on the aetiology of fatness in mammals (including humans). However they demonstrate the phenomenon of breed-specificity and thus highlight the need for the study of multiple populations to verify genotype - phenotype correlations.

## Methods

### Sample acquisition

An Aloka 500 Ultrasound scanner, coupled with AUSkey fat and muscle depth system software was able to provide an accurate representation of average fat depth (aFd) and average muscle depth (aMd) for 18757 Duroc pigs, 26992 Sire line large white and 27537 Titan (Pietrain) animals (this measurement is directly correlated with the total fat in the carcass [79]). Raw data was then converted to EBVs correcting the rib fat depth to the fixed animal weight of 91 kg. EBVs for each of the 70,000+ animals were obtained from the standard evaluation scheme employed by JSR using best linear unbiased prediction (BLUP) analysis. All samples were from males, with a similar genetic background and reared under identical conditions (stocking, density, feed, space etc.), in order to prevent other potential contributing factors that could influence any conclusions drawn. For selective genotyping, animals in the upper 5th percentile and the lower 5th percentile of the EBV range for fatness were taken forward for further investigation. All DNA samples were extracted from blood spots stored on FTA Whatman Cards™ that were sourced from the JSR Genetics (Driffield UK) archive. Of the 288 samples used, 96 were from each of the 3 aforementioned breeds; 48 from each group either in the upper or lower 5th percentile and their EBV values used for subsequent calculation. DNA isolation, amplification and SNP chip interrogation was performed using the method previously developed in house [80], with several alterations to the manufacturer’s instructions, including 2 punches being removed as opposed to 1, heating of the cards and washes being performed using water instead of FTA purification reagent and Tris EDTA. Extracted samples were amplified via Whole Genome Amplification (WGA) using the Sigma-Aldrich WGA2 kit, in order to produce an appropriate amount of DNA for microarray analysis, following manufacturer’s instructions. This fragmentation based WGA produces short 400-600 bp overlapping fragments that are primed with defined 3′and 5′ ends and amplified via linear amplification followed by geometrical amplification, therefore generating a thorough coverage of the genome.

### Genotyping

#### SNP analysis

Illumina Porcine SNP60 Genotyping BeadChips were interrogated with WGA amplified DNA from each porcine sample, according to manufacturer’s instructions, in an approach similar to that described by our own group [81,82] at Cambridge Genomic Services, Department of Pathology, University of Cambridge. All DNA concentrations were adjusted to a concentration of 50 ng/μl, in a final volume of 5 μl per sample. Raw fluorescence data were captured and normalized, using internal and external controls, and stored as image files. Following scanning, image data were transferred to the GenomeStudio Software framework V2010.1 and converted from fluorescence data to genotypic data based on the manufacturer’s design algorithms and the call rates produced by the Illumina software were determined. Significance analysis of the SNP chip data was performed, assuming an additive model and the raw data was corrected to the fixed animal weight of 91 kg, and then converted to EBVs using the PEST software. The model used herd/sex/season as a fixed effect with litter fitted as a random effect. The genotype scores for a given SNP were 1, 2 and 3 for genotypes AA, AB, and BB, respectively. For a given SNP only records were used when the genotype was known, any animals for which records were unknown were removed from the study. Analysis of variance was performed to obtain the P values. Due to multiple testing, Bonferroni corrections were implemented in order to determine the appropriate significance value. Due to multiple testing, an empirical genome wide significance threshold was calculated using permutation analysis, where SNPs are not considered to all be independent. Genotypes for all individuals were permuted and the GWAS analysis was calculated in all SNPs with the smallest p value being used to calculate the distribution. The permutation analysis was repeated 10,000 times and the value for the top 5% was deemed to be the significance threshold. SNPs that deviated from Hardy-Weinberg Equilibrium (HWE) were also removed, so results were not skewed. Significant SNPs were consequently investigated to determine whether they were located within, or close to a gene, using the Ensembl output for orthologous regions. This involved data mining using a combination of Ensembl (http://www.ensembl.org) and NCBI to interpret where the SNP resided (pig genome assembly version 9.2). A window of 1 Mb was examined (0.5 Mb upstream and 0.5 Mb downstream from the SNP was considered). If a SNP resided within a gene this was also noted. Subsequently, gene function was determined and SNPs were then placed into 2 groups, those in which their function suggested a pathway in which the control of fatness might be implicated, and those that were not. In order to determine the location and function of a SNP of interest within a gene, the variant effect predictor, a tool available from Ensembl was used (http://www.ensembl.org/info/website/upload/var.html).

#### CNV analysis

In order to derive CNV information from the existing SNP data, 2 analytical tools for the determination of copy number variation using SNP genotyping data were used, QuantiSNP and cnvPartition. QuantiSNP uses an Objective Bayes Hidden-Markov Model (OB-HMM) to estimate probabilities of CNV/aneuploidy detection [83]. QuantiSNP uses both LRRs and BAFs in order to call CNVs with corrections for differences in GC content also being employed in order to correct signal strength [84]. cnvPartition calculates copy number variants along with scores of confidence, therefore indicating the locations of CNV regions (http://www.illumina.com) using both BAFs and LRRs for each of 14 genotypes (double deletion, A, B, AA, AB, BB, AAA, AAB, ABB, BBB, AAAA, AAAB, ABBB, BBBB) as a simple bivariate Gaussian distribution. Samples with a call rate below 0.9 were removed, and the confidence thresholds and number of probes needed to determine a CNV event were altered accordingly; increasing the threshold improved the clarity of the output, while using a high probe count increased the stringency. 3 outputs were produced, 1 per breed with CNV fold change being represented in different colours for 5 groups (0–0.5, 0.5-1.5, 1.5-2.5, 2.5-3.5 and 3.5-4.5). Data derived from QuantiSNP graphs and cnvPartition outputs were subsequently collated. Only CNVs present in more than 5 animals for both QuantiSNP and cnvPartition were considered. The chromosome, the start and end of each of the CNVRs, the estimated copy number of the sequence and the number of animals in which each was observed (2 copies was considered to be typical as pigs are diploid organisms) were recorded. Mann Whitney U tests (2 tailed) were performed the data to determine if there were any significant differences between samples in the upper and lower 5th percentiles with p values of ≤0.05 being considered statistically significant.

## Abbreviations

aFd: average fat depth; aMd: average muscle depth; BAF: B allele frequency; BLUP: Best linear unbiased prediction; CNV: Copy number variant; CNVR: Copy number variable region; EBV: Estimated Breeding Value; FDR: False discovery rate; FCR: Food conversion ratio; GWA: Genome wide association; GWAS: Genome wide association study; HWE: Hardy Weinberg equilibrium; HSA: *Homo sapiens*; LRR: Log *r* ratio; MAF: Minor allele frequency; SNP: Single nucleotide polymorphism; SSC: *Sus scrofa*; T2D: Type II diabetes; WGA: Whole genome amplification.

## Competing interests

GAW and SW are employees of JSR genetics, who could, potentially, benefit financially from the results of this study.

## Author’s contributions

KF performed the majority of the experiments in the paper and co-wrote the manuscript. RPW analysed the raw SNP chip data. JB analysed the raw data from cnvPartition. EC, CR and NA performed the microarray experiments and generated the call rate data. SW coordinated all the samples for the project as well as the phenotyping. GAW and DKG conceived the project, supervised all aspects of it and co-wrote the manuscript. All authors read and approved the final manuscript.

## Supplementary Material

Additional file 1: Figure S1 Chromosome position of putative CNVs ascertained by quantiSNP and CNV partition. Left hand chromosome denotes result from CNV partition, right hand chromosome from quantSNP. Each putative CNVR is depicted as an elliptoid shape, colour coded for each breed as indicated. The numbers within the shape indicate the number of animals in with each putative CNV was found. If to the left of each chromosome a potential loss compared to the reference genome is apparent, a potential gain if to the right. Click here for file
