# Supplementary material for: Genome wide analysis reveals single nucleotide polymorphisms associated with fatness and putative novel copy number variants in three pig breeds
Source: BMC Genomics. 2013 Nov 13;14:784. doi: 10.1186/1471-2164-14-784 (PMC3879217; doi:10.1186/1471-2164-14-784)
Supplement: Additional file 1: Figure S1 — Chromosome position of putative CNVs ascertained by quantiSNP and CNV partition. Left hand chromosome denotes result from CNV partition, right hand chromosome from quantSNP. Each putative CNVR is depicted as an elliptoid shape, colour coded for each breed as indicated. The numbers within the shape indicate the number of animals in with each putative CNV was found. If to the left of each chromosome a potential loss compared to the reference genome is apparent, a potential gain if to the right. [file 1471-2164-14-784-S1.pdf]

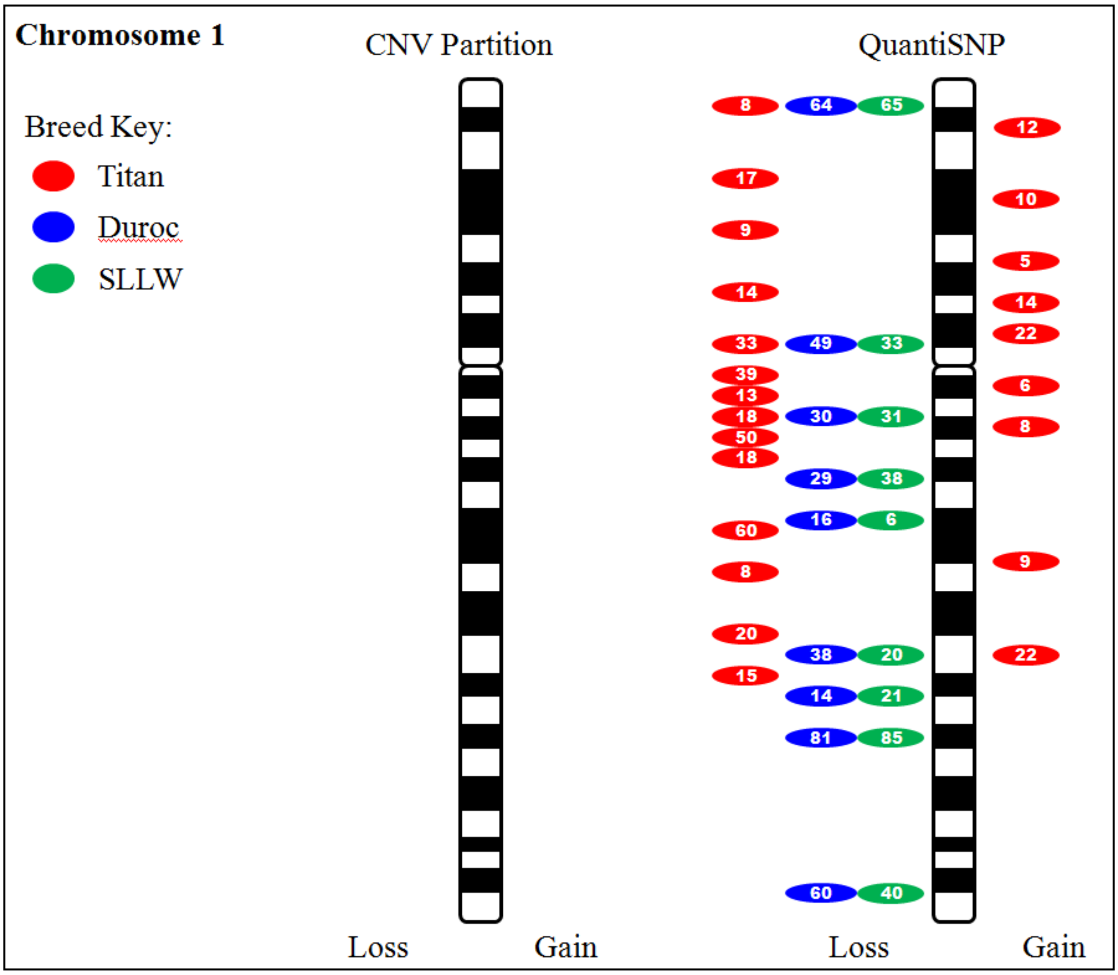
Chromosome 1
CNV Partition
QuantiSNP
Breed Key:
Titan
Duroc
SLLW
8 64 65
12
17
10
9
5
14
14
22
33 49 33
39
13
6
18 30 31
8
50
18
29 38
16 6
60
9
8
20
38 20
22
15
14 21
81 85
60 40
Loss
Gain
Loss
Gain

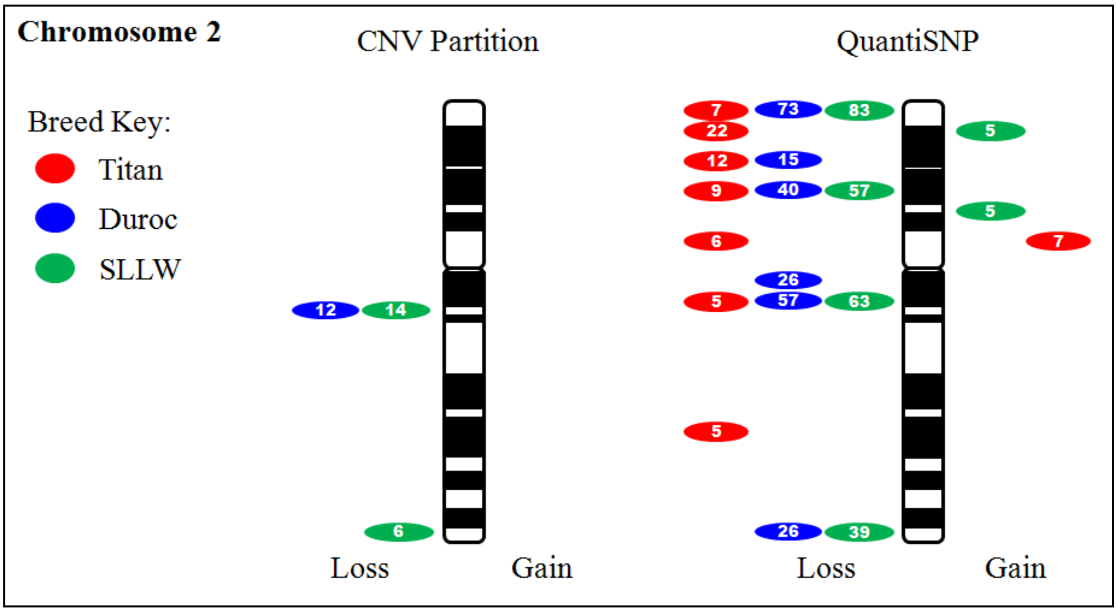
Chromosome 2
CNV Partition
QuantiSNP
Breed Key:
Titan
Duroc
SLLW
7 73 83
22
5
12 15
9 40 57
5
6
7
26
12 14
5 57 63
5
6
26 39
Loss
Gain
Loss
Gain

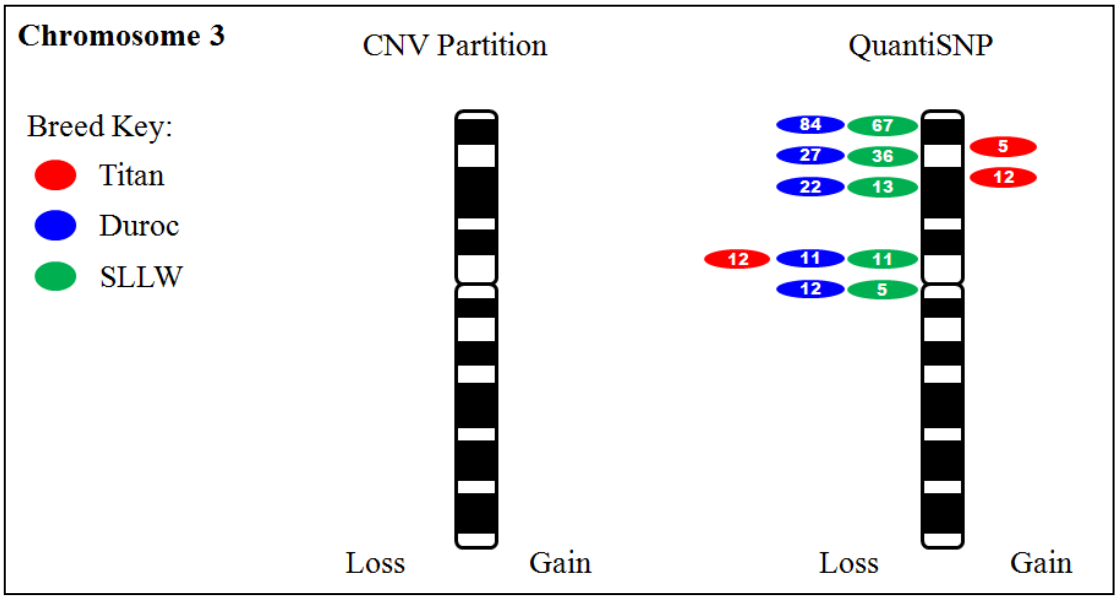
Chromosome 3
CNV Partition
QuantiSNP
Breed Key:
Titan
Duroc
SLLW
84
67
27
36
22
13
5
12
12
11
11
12
5
Loss
Gain
Loss
Gain

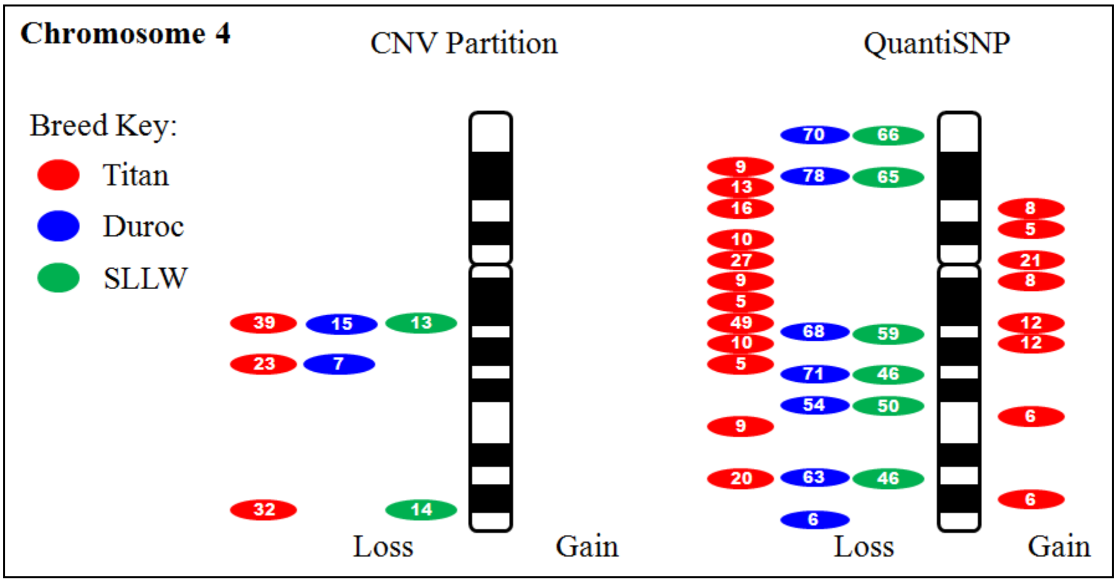
Chromosome 4
CNV Partition
QuantiSNP
Breed Key:
Titan
Duroc
SLLW
39
15
13
23
7
32
14
70
66
9
13
78
65
16
8
5
10
27
21
9
8
5
49
68
59
12
10
12
5
71
46
54
50
9
6
20
63
46
6
6
Loss
Gain
Loss
Gain

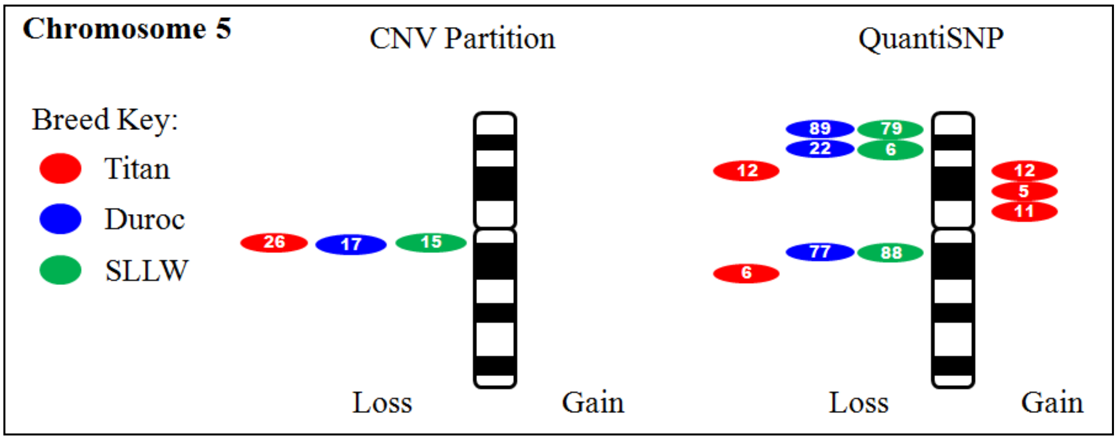
Chromosome 5
CNV Partition
QuantiSNP
Breed Key:
Titan
Duroc
SLLW
26
17
15
89
79
22
6
12
12
5
11
77
88
6
Loss
Gain
Loss
Gain

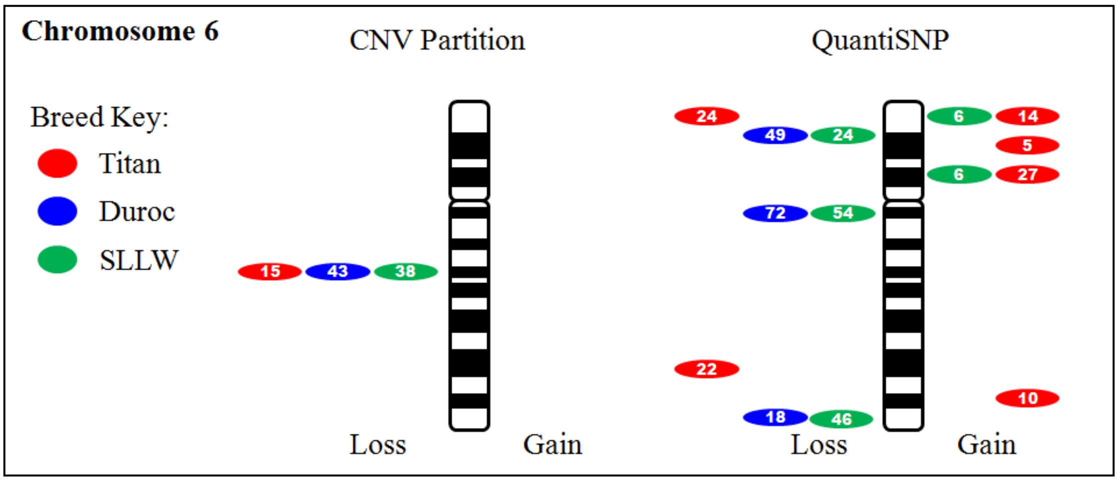
Chromosome 6
CNV Partition
QuantiSNP
Breed Key:
Titan
Duroc
SLLW
15
43
38
24
49
24
6
14
5
6
27
72
54
22
10
18
46
Loss
Gain
Loss
Gain

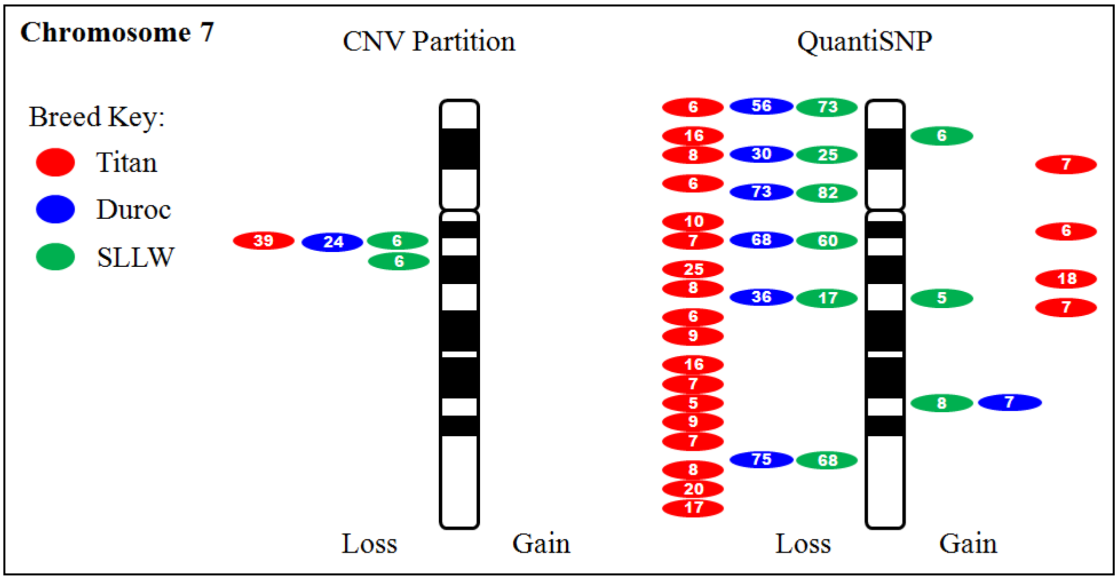
Chromosome 7
CNV Partition
QuantiSNP
Breed Key:
Titan
Duroc
SLLW
39
24
6
6
6
56
73
16
8
30
25
6
73
82
10
7
68
60
25
8
36
17
6
9
16
7
5
9
7
8
20
17
75
68
6
7
6
5
18
7
8
7
Loss
Gain
Loss
Gain

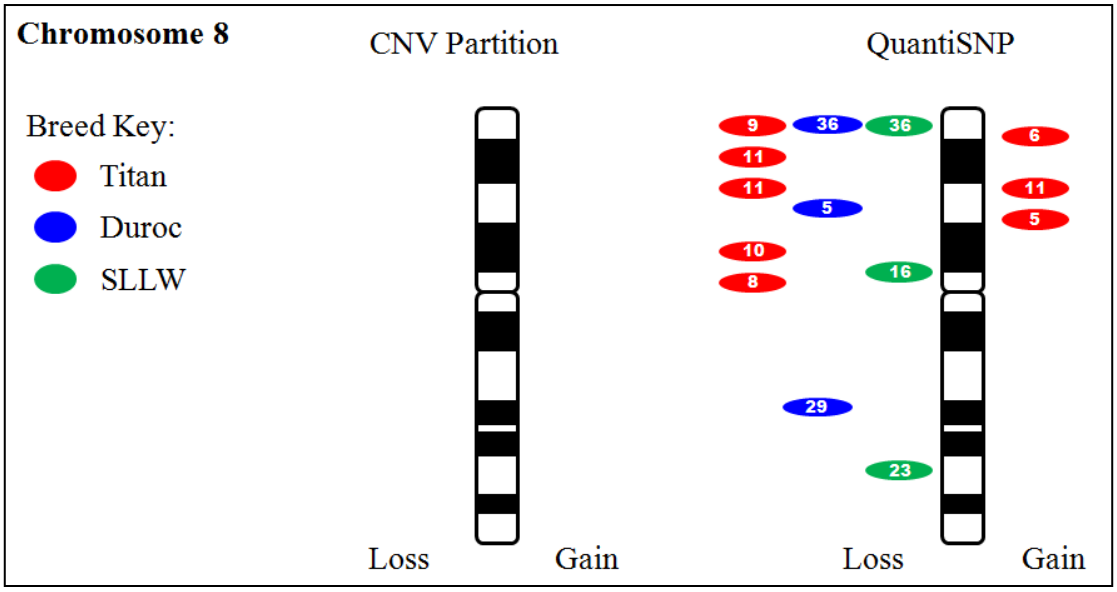
Chromosome 8
CNV Partition
QuantiSNP
Breed Key:
Titan
Duroc
SLLW
9
36
36
11
11
5
10
8
16
29
23
6
11
5
Loss
Gain
Loss
Gain

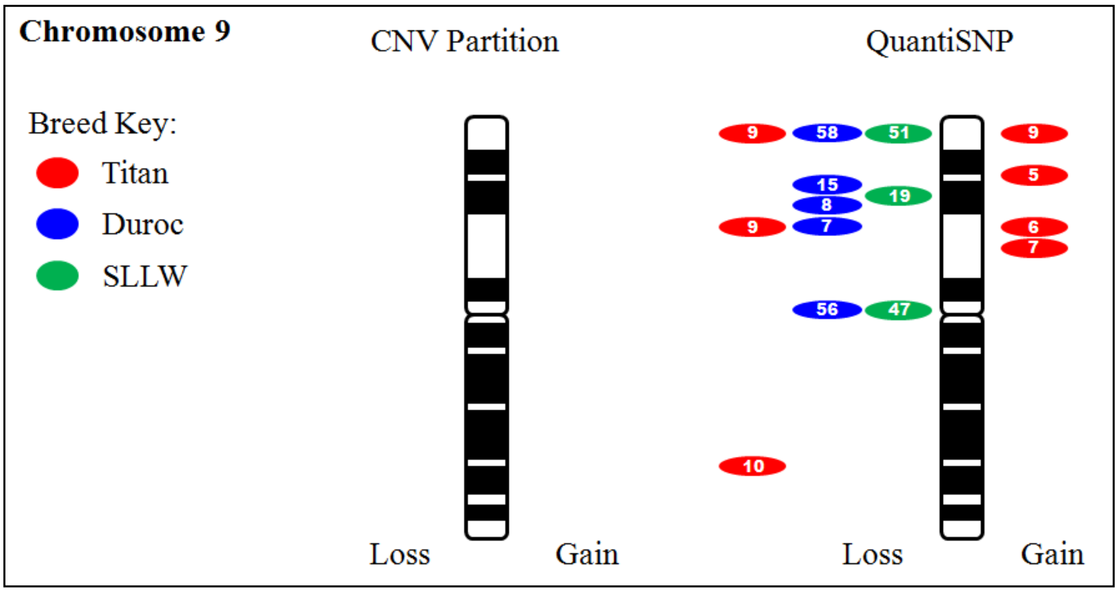
Chromosome 9
CNV Partition
QuantiSNP
Breed Key:
Titan
Duroc
SLLW
9
58
51
9
15
19
5
8
9
7
6
7
56
47
10
Loss
Gain
Loss
Gain

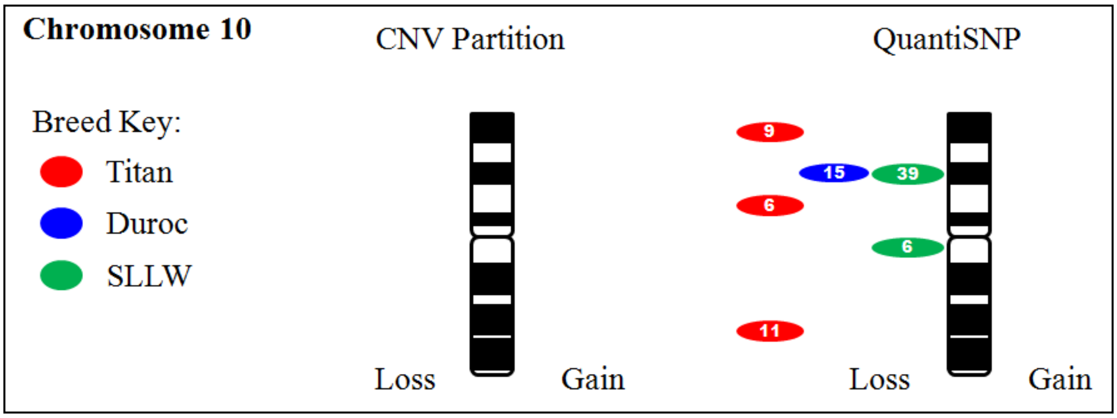
Chromosome 10
CNV Partition
QuantiSNP
Breed Key:
Titan
Duroc
SLLW
9
15
39
6
6
11
Loss
Gain
Loss
Gain

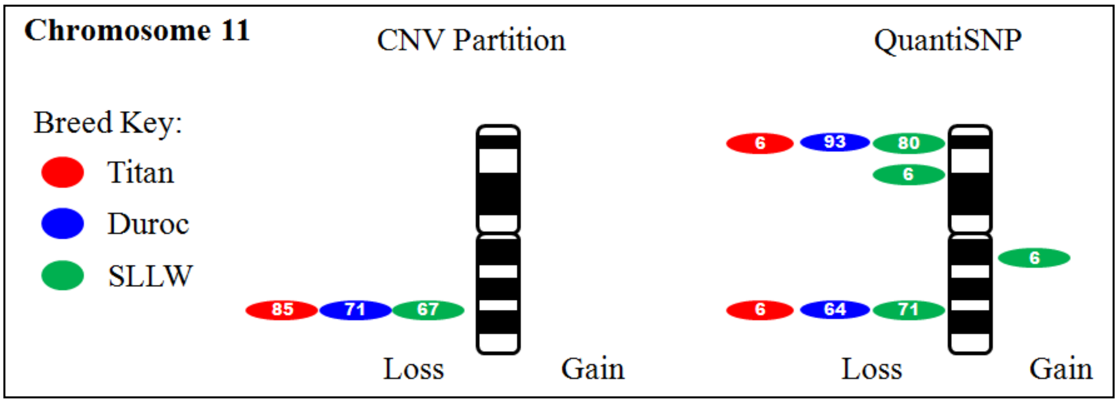
Chromosome 11
CNV Partition
QuantiSNP
Breed Key:
Titan
Duroc
SLLW
6
93
80
6
6
85
71
67
6
64
71
Loss
Gain
Loss
Gain

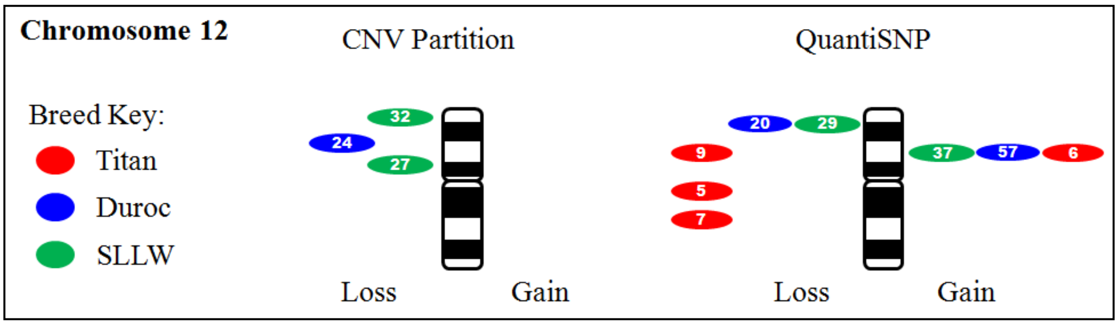
Chromosome 12
CNV Partition
QuantiSNP
Breed Key:
Titan
Duroc
SLLW
32
24
27
20
29
9
5
7
37
57
6
Loss
Gain
Loss
Gain

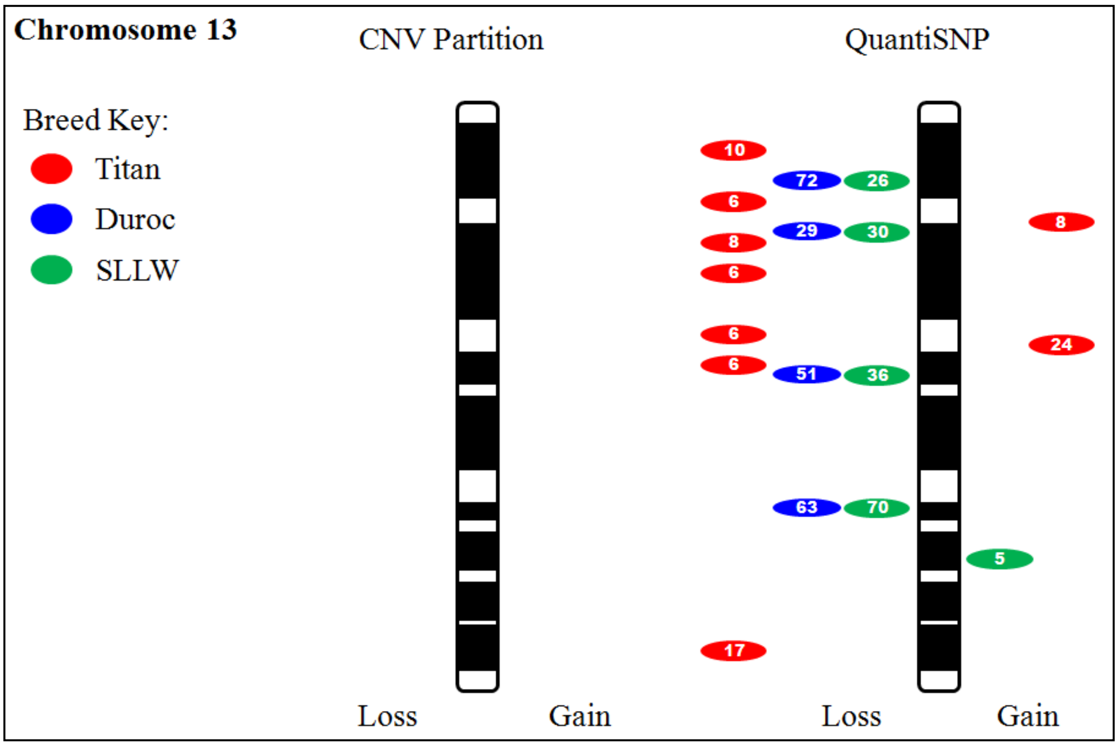
Chromosome 13
CNV Partition
QuantiSNP
Breed Key:
Titan
Duroc
SLLW
10
72
26
6
29
30
8
6
8
6
24
6
51
36
63
70
5
17
Loss
Gain
Loss
Gain

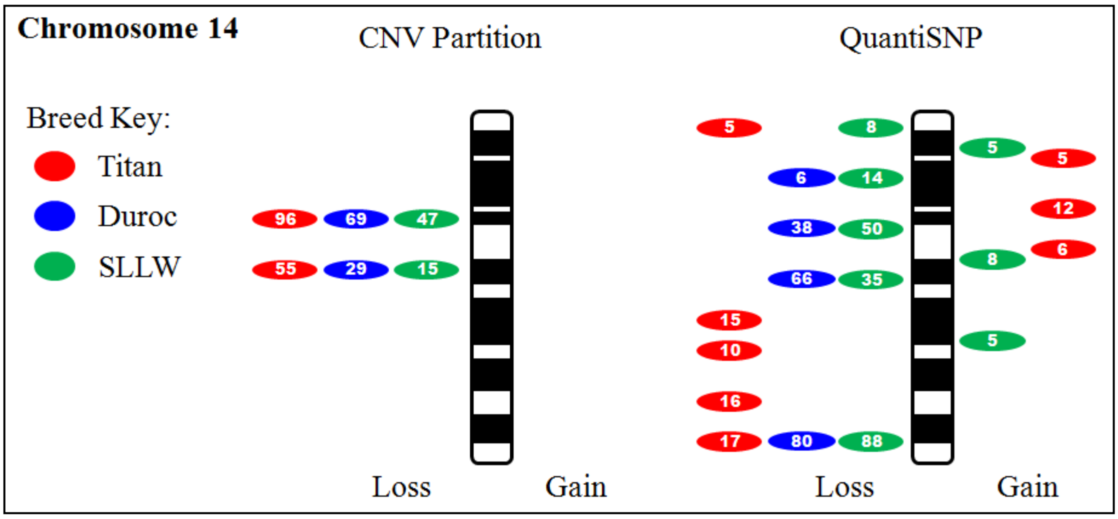
Chromosome 14
CNV Partition
QuantiSNP
Breed Key:
Titan
Duroc
SLLW
96
69
47
55
29
15
5
8
5
5
6
14
12
38
50
8
6
66
35
15
5
10
16
17
80
88
Loss
Gain
Loss
Gain

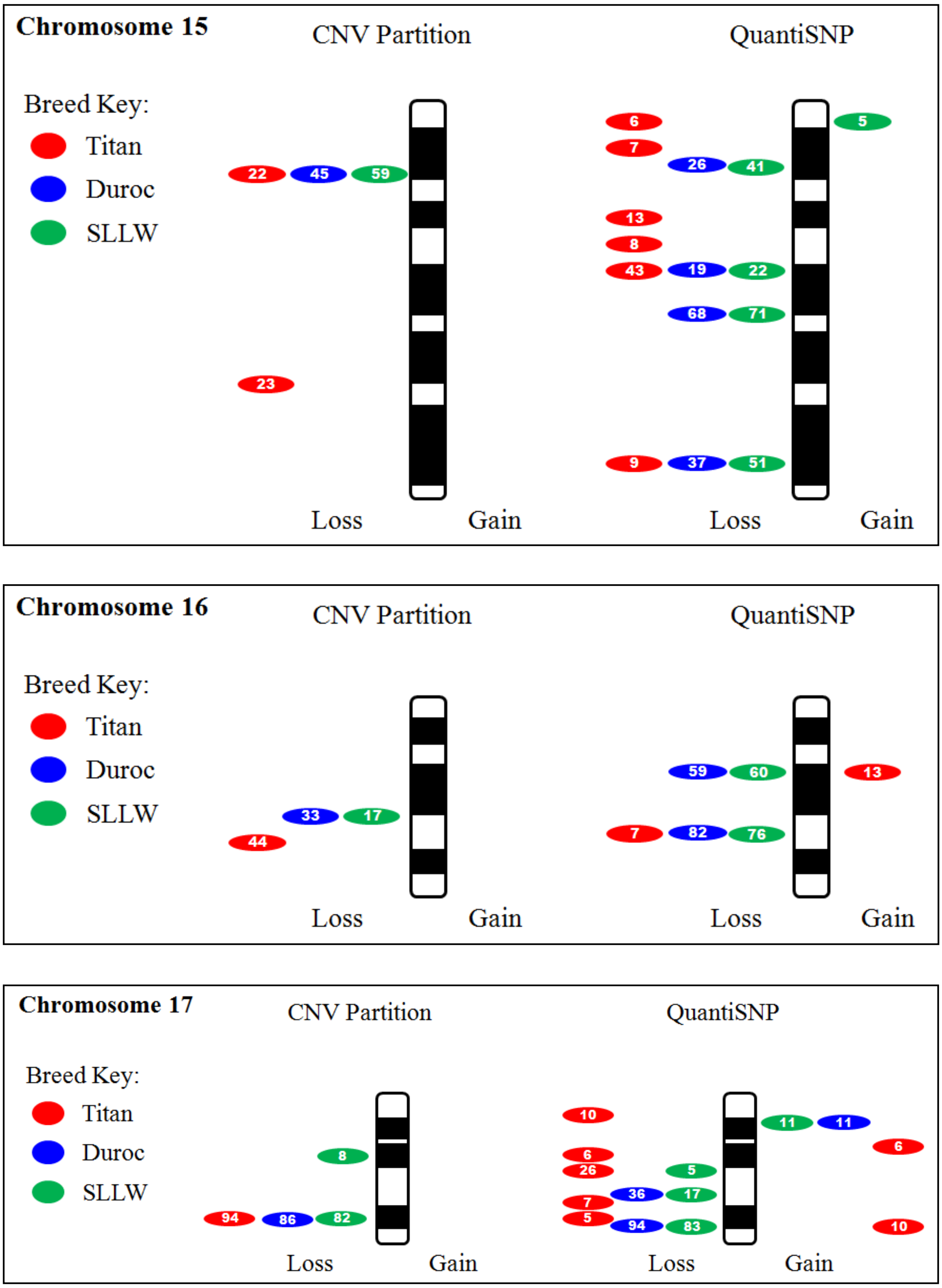

Chromosome 15
CNV Partition
QuantiSNP
Breed Key:
Titan
Duroc
SLLW
22 45 59
23
Loss
Gain
6
7
5
26 41
13
8
43 19 22
68 71
9 37 51
Loss
Gain
Chromosome 16
CNV Partition
QuantiSNP
Breed Key:
Titan
Duroc
SLLW
33 17
44
Loss
Gain
59 60
13
7 82 76
Loss
Gain
Chromosome 17
CNV Partition
QuantiSNP
Breed Key:
Titan
Duroc
SLLW
8
94 86 82
Loss
Gain
10
11 11
6
6
26
5
36 17
7
5 94 83
10
Loss
Gain

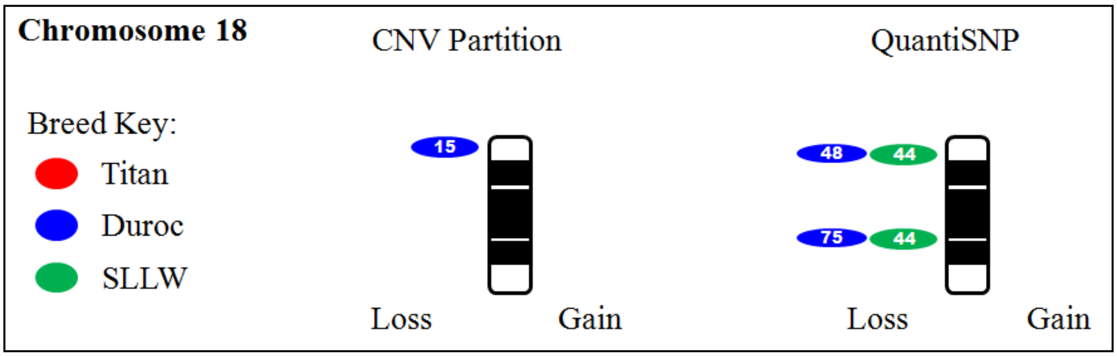
Chromosome 18
CNV Partition
QuantiSNP
Breed Key:
Titan
Duroc
SLLW
15
48
44
75
44
Loss
Gain
Loss
Gain

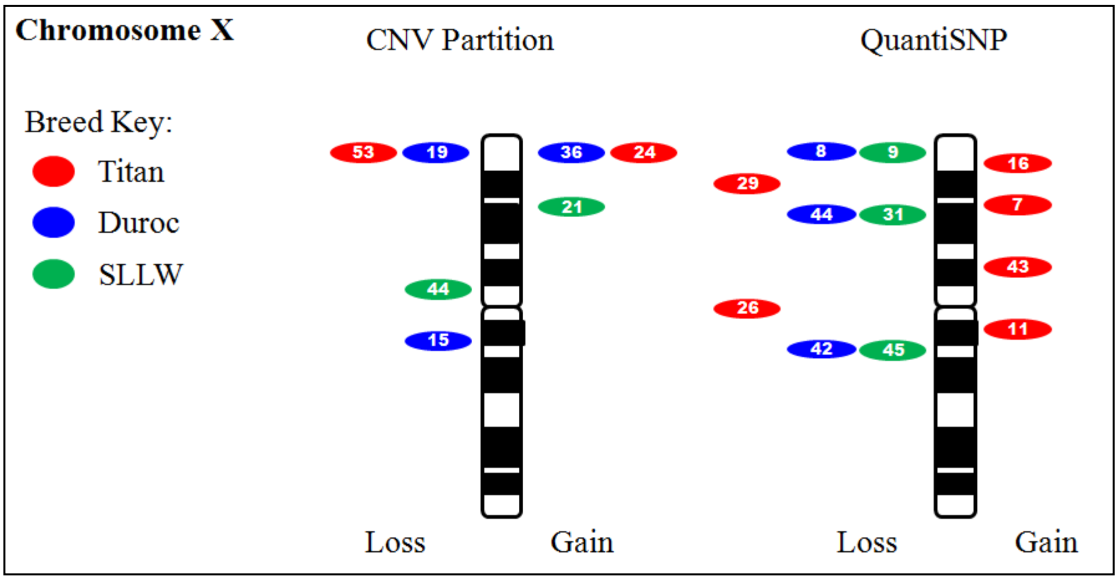
Chromosome X
CNV Partition
QuantiSNP
Breed Key:
Titan
Duroc
SLLW
53
19
36
24
21
44
15
8
9
16
29
44
31
7
43
26
11
42
45
Loss
Gain
Loss
Gain
